# Treatment of Exposed Tooth-Pulps, with a View to Preservation

**Published:** 1889-11

**Authors:** James S. King

**Affiliations:** Pittsburg


### TREATMENT OF EXPOSED TOOTH-PULPS, WITH A VIEW TO PRESERVATION.[1]

BY JAMES S. KING, D.D.S., PITTSBURG.

I HAVE concluded that I could the more intelligently and understandingly present the sum of my thoughts and the small accumulations gathered from my experiences in relation to the subject I have chosen by giving in brief detail a definite statement of the manner of treating exposed tooth-pulps which for almost a score of years I have employed in my practice. I also purpose giving a brief account of the method of treatment I have been pursuing in those cases in which the tooth-pulps are on the verge of that condition called absolute exposure.

In order to attain an average success in operations which have for their ultimate object the preservation of vitality in exposed tooth-pulps, the first step to be taken is to provide the needed remedies and materials. In my practice these are few in number, —viz., wood creosote, zinc oxide, iodoform, paper treated in zinc chloride, and crystalline cement. A correct rule of practice in the treatment of exposed tooth-pulps is that all necessary preparations of the cavity of decay should be brought to a finish, with rubber-dam in place prior to placing within the cavity the materials intended for the protection of the pulp. In preparing the remedies and materials I have named for use in pulp treatment, my first move is to combine with wood creosote a portion of zinc oxide in amount sufficient to form a somewhat stiff paste. Then the rubber-dam being in place and the cavity properly prepared, I take a general view of the exposure and surroundings, in order to determine how large or how small I shall cut the fragment of paper for immediate use, the diameter of which should be such as to permit of an overlapping to some extent of the dentine that forms the border of the exposed orifice. Then on one side of the paper I put a small portion of creosote paste, and with a pair of delicately pointed foil-pliers seize the paper, apply that side to the point of exposure, and in a gentle manner press the paper into the desired position, being careful to prevent the formation of air-bubbles. I now proceed to cover paste and paper with a coating of phosphate

[1] Read at the twenty-first annual meeting of the Pennsylvania State Dental Society, held at Cresson, Pa., July 31, 1889.

cement. Recently a friend suggested to me the use of Weston's non-irritant cement, as being a better article on account of its non-irritating qualities. On either of these cements any character of filling can be placed, thus completing the operation. Much depends on a correct diagnosis of each individual case, prior to engaging in the labor of pulp-treatment. Any case presenting undoubted evidences of inflammation and congestion or strangulation of the pulp should not be treated in the manner I have just pointed out.

A few words now in relation to cases in which absolute exposure of the pulp has not taken place, cases in which the film or septum of dentine remaining in place between the cavity of decay and the pulp-tissue has become very thin and sensitive. This condition is often met with in practice, and in some instances it causes much annoyance and discomfort to the patient subsequent to the operation, unless the prospective trouble is properly guarded against prior to the insertion of a metallic filling. It is well known to all who have had an average experience in dental practice that tooth-pulps not actually exposed do in some instances lose their vitality, as a consequence of their nearly exposed condition. This result in some instances may arise from the action of the thermal changes that are ever occurring in the bodies of large metallic fillings. In all cases in which I am led to believe or even to suspect that there exists an unsafe condition of thinness in the dentine between the cavity of decay and the pulp-tissue, I at once make use of creosote paste and chloride paper. I cut the paper in such form and size as will permit a little overlapping of the margin of the thin dentine, thus affording ample protection to the thin septum of dentine and to the underlying pulp. When it is my purpose to finish an operation by the use of gold, I place over the paste and paper a portion of phosphate cement, to serve as a base for my filling. In some cases, however,—for instance, a superior lateral incisor, or inferior central or lateral, in which there is not sufficient space,—I do not use the cement. I apply the gold in direct contact with the paper. I do the same in some instances in which I have absolute exposure of the pulp, and also in a majority of cases in which I use amalgam. I employ cement only in large cavities wherein pulp-exposure is correspondingly large. Cement is required in order to avoid undue compression of the pulp. Frequently cases are met with in which there is present a high state of inflammatory action and congestion of the pulp-tissue, and while actual strangulation of the pulp has not taken place, yet the vital forces are seemingly crowded near to the verge of dissolution, when possibly there may be inherent within the body of such pulp a vitality sufficiently strong and active to respond to the action of the restorative and curative agents made use of. If in my judgment I have before me a combination of symptoms like those I have enumerated, I at once make ready the cavity for treatment of the pulp, by removing the decomposed tooth-substance, and washing the cavity with a pledget of cotton wet in wood creosote. I now combine with creosote paste a little iodoform, and, applying the mass to the exposed pulp as in ordinary cases, fill the remainder of the cavity with cement, and dismiss the patient for two months to await results.

